# Understanding the psychological impact of flooding on older adults: A scoping review

**DOI:** 10.1111/nyas.15356

**Published:** 2025-05-14

**Authors:** Sarah Law, Temenuzhka Marinova, Lillie Ewins, Elizabeth Marks

**Affiliations:** ^1^ Department of Psychology University of Bath Bath UK

**Keywords:** climate change, elderly, flooding, mental health, older adults, review

## Abstract

Flooding is increasing in frequency and intensity as a function of climate change. Older populations are vulnerable to the physical and mental health impacts of flooding, having less ability to cope and fewer rescue possibilities compared to younger ages, with resulting mental health impacts. This scoping review, based on JBI Scoping Review Methodology, scoped and summarized the evidence for the psychological impacts of flooding on older adults. Electronic databases were searched for reports of direct or indirect experiences of flooding in older adults globally (mental health, well‐being, emotional outcomes). Ten articles across four continents were included. Risk of bias analysis found that 80% of articles were of weak and 20% were of moderate quality. Most studies focused on depression (70%), posttraumatic stress (60%), and anxiety (20%) as outcomes. Over half considered additional impacts and protective factors. This small but growing literature base demonstrates that climate‐related flooding affects the mental health of older adults, with associations between flooding, depression, and posttraumatic stress. Poor social support contributes to worse mental health outcomes, suggesting that helpful interventions might focus on enhancing resilience through building social networks. All studies reviewed were from high‐income countries; more research is required with countries with lower income.

## Graphical abstract

This scoping review assessed quantitative literature in 10 articles across 4 continents, exploring the mental health impacts of flooding on older adults. Climate change is increasing flooding severity and frequency, and older populations may be particularly vulnerable. Findings indicated that climate‐related flooding is associated with depression and posttraumatic stress in older adults. Poor social support contributes to worse mental health outcomes. More research is required, particularly in low‐income countries.

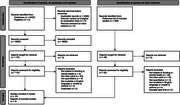

## INTRODUCTION

“Climate change presents a fundamental threat to human health”^1^ and to societies across the world.^2^ Climate change leads to weather extremes and climate anomalies worldwide, with widespread loss and damage to people and the environment. This will escalate with predicted increases in global temperatures.^3^ Roughly half of the world's population resides in areas highly vulnerable to the effects of climate change, and there has been a 15‐fold increase in fatalities from floods, droughts, and storms over the last decade.^3^ The physical health consequences of extreme events are mediated through social systems (e.g., disrupted health services) and environmental systems (e.g., increase in water‐borne diseases post‐flood).^4^ Such consequences are exacerbated by having limited evidence on effective public health interventions to support welfare following climate events.^5^ Some evidence suggests existing services and governmental initiatives are ill‐equipped to cope.^6^

The direct and indirect effects of climate change increase vulnerabilities to physical and mental health conditions via multiple pathways: damaging livelihoods, preventing access to health care, reducing social support structures, spreading disease, and increasing mortality risk.^7^ Climate change can have enormous impacts on mental health outcomes^8^; for example, individuals directly impacted by extreme weather events are more likely to develop posttraumatic stress disorder (PTSD), depression, or extreme distress than those without such.^2^ Again, such outcomes will likely worsen as climate change increases the risk of extreme weather events.^6^

Globally, flooding is one of the most prevalent disasters related to climate change.^9^ It represents the greatest cause of human displacement compared to other natural disasters.^10^ Flooding is predicted to become more frequent and intense with the warming planet^3^; projections indicate that the number of individuals displaced by flooding will double by the end of this century.^11^ Flooded areas with inadequate infrastructure and limited financial resources experience the greatest levels of human mortality.^5^ Individuals directly affected by flooding report greater rates of climate change concern than those not directly affected,^12^ and significant psychological impact is reported in individuals who experienced repeated flooding causing property damage,^2^ indicating how direct exposure to climate disasters could be associated with eco‐distress.

Alongside the growing impacts of climate change, the global population is also aging. Countries around the world are seeing a greater number of citizens aged 60 years and over, and demographic data indicate this will continue for decades.^7^ Aging is associated with biopsychosocial complexities that can increase vulnerability to mental health challenges. For example, aging has been associated with increased levels of discrimination and negative attitudes toward older generations.^13^ Such ageism can put the psychological health of older adults at risk^13^ and may be particularly exacerbated at times of societal stress. For example, following the COVID‐19 pandemic, reports indicated that older adults faced mistreatment, hate speech, and were given the lowest priority for life‐saving treatment.^14^

The impacts of climate change–related disasters can permeate multiple areas of life. Older adults appear particularly vulnerable to consequences such as increased risk of suicidality, insomnia, substance abuse, behavioral disturbances, and cognitive difficulties.^15^ After sudden‐onset natural disasters, and when compared to younger populations, older adults were more likely to experience symptoms of PTSD and adjustment disorder.^16^ Older adults may also have a particular relationship to the climate crisis itself based on their lifespan. The worst consequences of climate change are developing now and are based on decades of fossil fuel emissions (particularly by wealthy countries), which has implications in terms of perceived responsibility. Older people could therefore experience a sense of guilt and powerlessness about climate change based on how they perceive their generation's contribution to climate change over the course of their lives.^17^

Flooding is of a particular concern as reports suggest older adults are less able to cope with flood waters, have less rescue possibilities, and so can feel more helpless than other age groups.^18^ Older age is associated with higher rates of physical disabilities, poor health, and cognitive changes. These impact mobility and hence pose evacuation challenges. Furthermore, higher rates of digital exclusion in older populations, alongside potential cognitive limitations, can limit access to the early warning procedures essential for improving outcomes in response to extreme weather.^9^ Together, this can result in greater risk of injury and death, for example, by drowning in flood water.^19^ Worse evacuation outcomes, lower awareness of dangers, and poorer access to emergency resources can lead older adults to be overlooked by emergency services far more than the general population.^20^

Older populations may depend more on support from others for safe evacuation following flooding, particularly if they have limited mobility, and this may be worsened if the resources of those upon whom they depend on are also compromised in some way (e.g., if their support networks are also impacted by flooding or are also older adults).^9^, ^19^ Access to appropriate transport can ensure self‐reliant evacuation opportunities, but a study on older adults in a Welsh coastal town found that many older adults lack personal transportation, making them more reliant on others to evacuate.^9^

Good access to social networks plays a role, and a study of 43 developing countries reported higher rates of older adults living alone when compared to the general population (8.8% vs. 1.6%, respectively).^21^ In the United Kingdom, almost 50% of those aged 75+ live alone.^22^ People living alone may find it harder to prepare to protect themselves prior to or during a flood event, and to recover post‐flood, all of which will be exacerbated if they also have health or mobility needs.^22^ Evidence indicates that older people may receive less social support post‐disaster, with negative consequences for physical and mental health outcomes.^17^ This is even more important in low‐ and middle‐income countries (LMICs), which may have more exposure to flooding and less resources to mitigate and respond to it on a population level. Recognition of specific vulnerabilities in older adults has been reflected in recent legal challenges, and in 2024, older women from Switzerland won the first ever climate case in the European Court of Human Rights. This recognized the failings of the government to mitigate and respond to climate change adequately, including in protecting older women from potentially life‐threatening vulnerabilities to the effects of climate change.^23^

Overall, older adults appear to have specific vulnerabilities to the impacts of flooding on their health, coupled with some lack of important protective factors. Most literature has focused on the physical health impacts, despite there being clear direct and indirect pathways to worse mental health outcomes in this group. As flooding increases around the globe, there is an urgent need to understand how flooding affects the mental health of older adults, which in turn could identify important targets for mitigation and adaptation. In response to this need, this study aimed to scope the existing literature on flooding and mental health in older adults, to improve the understanding of the state of the existing literature, to demonstrate what is already known, and to identify areas for future research.

In line with previous scoping reviews of climate‐related disasters and mental health,^24^, ^25^ and based on recent scientific consensus,^3^ there are clear links between climate change and the increase in significant flood events in recent years. It is important to note that the complex nature of weather makes directly attributing a specific flood to climate change challenging, although there are clear arguments that climate change increases the likelihood of storms and flooding significantly. Recent systematic reviews of all age groups have discussed flood events as climate events for a similar reason, although some included articles have not identified such floods as directly caused by climate change.^26^, ^27^ Such studies still provide important information about the impact of flooding on mental health, which will be of vital import as flooding increases in severity and frequency in the coming decades. The current review is a response to growing recognition of the importance of this area, the increasing amount of flooding, the aging global population, and the lack of a specific focus on this important age group with specific needs.

### Objectives

This scoping review and narrative synthesis aimed to provide a comprehensive picture of the current state of the evidence relating to the mental health impacts of flooding that is likely related to climate change on the older adult population. The overarching objective of this scoping review was to address this gap in the literature by exploring the psychological impact of flooding on older adults globally, with three specific aims:

- To identify and provide a comprehensive picture of the quantitative evidence into the mental health impacts of flooding on older adults.
- To describe the characteristics of studies, including the methodological approaches employed, and review the quality of this literature.
- To identify gaps in the current knowledge base to inform future research and initiatives.

## MATERIALS AND METHODS

This review followed the JBI Scoping Review Methodology.^28^ The protocol for this review was registered as a larger study on Open Science Framework (https://osf.io/e32gj). This larger study aimed to explore the mental health impacts of climate change on older adults, which resulted in a very large number of papers being retrieved (*n* = 9459). The research team discussed this outcome and concluded that a more helpful synthesis would involve focusing scoping questions on particular aspects of climate change impacts on older adult mental health. The review was therefore divided into specific topics, which could each allow for a more meaningful synthesis of each aspect of climate change in this population. The initial selection of climate change–related events was determined by reviewing the IPCC, WHO, and UN websites, and flooding related to climate change was identified as representing the greatest prevalence and disruption globally.^9^, ^20^ This, alongside existing evidence for the probable increased vulnerability that older adults face from the impact of flooding,^18^ shaped the focus of this scoping review.

### Search strategy

The search strategy drew upon insights from previous reviews and was developed by the research team and department librarian. Search terms were developed from reviews on the impact of climate change on mental health^8^, ^29^, ^30^ and older adults.^31^ The following databases were searched between July 2023 and December 2023: Cochrane Database of Systematic Reviews, PubMed, APA PsycNet, Embase, Web of Science (including Web of Science Core Collection, Korean Journal Database, and SciELO Citation Index), GreenFILE, and Scopus. Unpublished and gray literature were searched using PsychExtra (via APA PsycNet) and PsychInfo/ ProQuest Dissertations and Theses (via Web of Science). Five authors (S.L., N.M., L.E., S.J., and P.W.) conducted title and abstract screening using Covidence and a Microsoft Excel spreadsheet to initially identify studies that were particularly focused on the mental health impacts of climate change events. From this, the papers relevant to this current scoping review on flooding were identified by two authors (S.L. and L.E.), who conducted full text screening of items describing the impact of flooding on mental health, as well as screening items that described other climate events that may describe the impact of flooding, such as hurricanes and storms, using a Microsoft Excel spreadsheet. The reference lists of all identified studies were manually scanned to identify any further relevant articles for the review. The search strings for each database can be found in Supporting Information S1. This review set a date limit of the year 2000 onward for inclusion of papers, and it is important to note that the IPCC had not acknowledged the effects of climate change on human well‐being prior to 2007.^24^ This year was decided to best reflect current understandings of the mental health impacts of climate change; this is in line with other reviews in this area^8^, ^30^ and the increasing awareness of climate distress from the 21st century.^32^

### Screening

The inclusion criteria were as follows:

#### Population

Data for individuals over 60 years of age were originally set as the target population. However, in reviewing the literature, the research team noticed that multiple studies referred to individuals over 55 years as older adults, with justification that this age range defines a life stage that in some countries is regarded as specifically relevant to later life. During the full text review, the team noted that the cut‐off age for older adults was often culturally determined. With an aim to be inclusive of global populations, the decision was thus made to adjust the inclusion criteria to 55 years or above. In studies where the sample was mixed, to meet the inclusion criteria they must have had either (a) older adults (aged 55+) representing more than 50% of the sample or (b) clearly separated age subgroups in the results that could be assessed independently for this review.

#### Concept

To be included in the review, articles needed to report on both flooding and direct or indirect psychological and mental health effects from such flooding. As described by Berry et al.,^33^ direct effects included direct exposure to flooding (e.g., as a traumatic event), and indirect effects included impacts on physical health from issues such as water‐borne disease, disruption to food supply or drinking water, and community well‐being impacts through economic stressors, or social isolation. Since the IPCC^3^ reported that it is likely climate change that has been the main driver behind the increased heavy rainfall, floods, and storms globally, and that this is set to worsen in the future,^24^, ^26^, ^27^ this review took the same stance as similar reviews. Thus natural disaster–related floods were included but cases where flooding was clearly related to different phenomena (e.g., a pipe‐burst) were excluded.

Any outcomes relating to mental health, well‐being, emotions, or concerns were included in this review. Studies were also included if they discussed psychological protective or vulnerability factors relating to flooding or resilience. Research that reported multiple climate events, where flooding was reported as a subgroup that could be identified and from which data could be extracted, were included.

#### Context

All geographical locations globally were included. However, due to restrictions of time and resource, research was only included if it was written or translated into the English language.

#### Sources

Only studies with quantitative methodologies were included in this review. Research that included qualitative research, meeting abstracts, letters, data papers, book reviews, news items, and retracted publications were excluded. While qualitative studies are important in exploring experiences, such studies can be less generalizable or representative of wider populations. As quantitative studies typically have larger sample sizes, it was deemed that prioritizing the inclusion of quantitative studies would be best‐fit to systematically identify and summarize the available evidence regarding the prevalence, patterns, and associations of the mental health outcomes of older adults related to the specific climate‐related disaster of flooding. This use of quantitative studies provided insight into the extent and scope of the impact of flooding on the mental health of older adults, which provides a helpful foundation for future systematic reviews.

**FIGURE 1:**
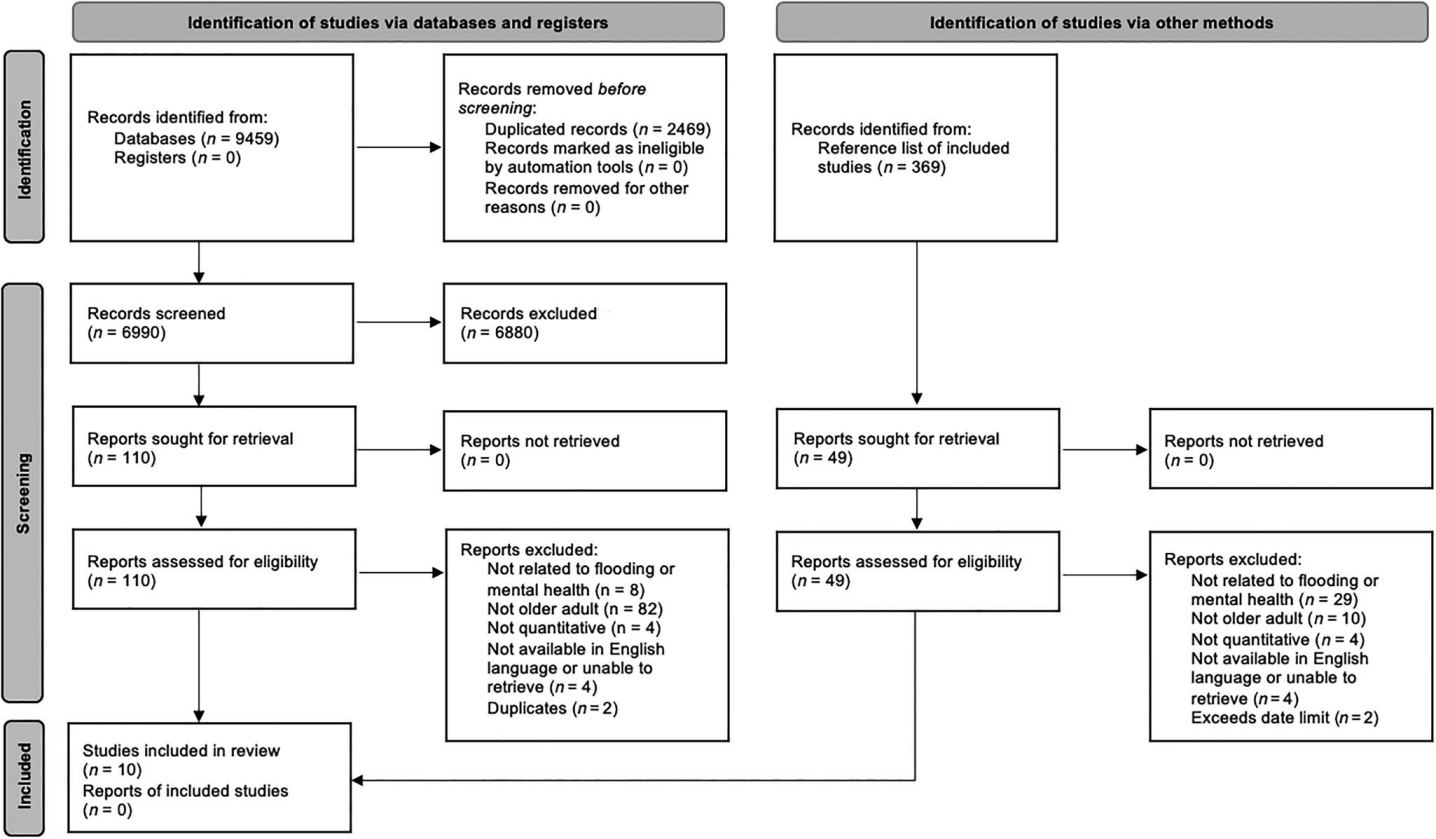
PRISMA flow diagram of study selection.

The PRISMA 2020 flow diagram (Figure 1) shows included and excluded studies.^34^ In total, 110 papers were identified from the initial searches that examined the direct and indirect impact of flooding on older adult mental health. The reference list of the 10 included articles was scanned manually to identify any further relevant articles for inclusion in the review; 49 additional articles were identified and reviewed for inclusion; however, no further papers met the inclusion criteria. Where disagreements arose, the reviewers discussed their assessments and reached a consensus.

### Data extraction

An extraction tool was developed by the reviewers to include information on the sample, study location, flooding event, study aims and design, findings relating to mental health impact, and future recommendations. The data extraction table headings can be found in Table S1.

### Quality assessment

Two of the authors (S.L. and L.E.) assessed all studies for quality using the Quality Assessment Tool for Quantitative Studies.^35^ Studies were assessed as *strong*, *moderate*, or *weak*, guided by the questions set by this tool. No articles were excluded due to their quality, as is customary with a scoping review.

## RESULTS

A total of 10 articles met the inclusion criteria. The inter‐rater reliability between the two authors was evaluated using Cohen's kappa coefficient (*k* = 0.780, *p* < 0.05). The percentage of observed agreement was 96.4%, indicating excellent agreement across raters. The data from each article were extracted by two authors (S.L. and L.E.) and cross referenced to ensure all data were captured. The extracted data included study characteristics, participant characteristics, information on flooding event, key findings pertaining to mental health outcomes along with additional outcomes, and future recommendations. Table 1 summarizes the included studies' key characteristics.

**TABLE 1:** Study characteristics.

	Study (author date, country)	Design, method	Sample *n*; characteristics; how older adults defined; recruitment method	Evaluation measures; mental health condition	Key findings	Future recommendations
1	Bei et al. (2013)^46^ Australia	Cohort study 10‐item online survey collected 2–7 months pre‐flood and 3–7 months post‐flood	274; Mean age 71.69 (SD = 7.86) Female (71.7%) Australian (93.3%) Retired (64.8%) Married/in relationship (80.9%) Religious (85.2%); Aged 60+; Convenience sampling	Impact of Event Scale—Revised, Geriatric Anxiety Inventory Centre for Epidemiological Studies Depression Scale, Satisfaction with Life Scale, Liverpool Stoicism Scale, Brief COPE; Trauma, Anxiety, Depression	Individuals who were personally affected by the floods scored higher on the Impact of Events Scale—Revised and greater increase in anxiety compared to nonaffected groups. The floods were found to have made a small but measurable impact on mood and satisfaction with life. Age, gender, employment status, and prior flood experience were found to be neither risk nor protective factors for well‐being. Greater exposure to floods (routine disruption, evacuation, financial loss) was associated with significantly worse mental health outcomes. Higher levels of PTSD symptoms were reported by those who received government and community support than those that did not. It is likely that the government support was provided to those who were more adversely affected accounting for the higher PTSD symptoms. Those with government and community support only reported worse physical health and satisfaction with life, compared to individuals with personal connections too. Transportation issues was problematic for older adults, suggesting those without good person support might be adversely affected by transportation disruption.	Effective tools are needed to identify individuals with symptoms requiring clinical attention for timely intervention and support. A proactive approach with this population would be helpful as older adults may not initiate help‐seeking. Strategies such as encouraging coping strategies such as acceptance, positive reframing, and humor could be particularly helpful.
2	Chen et al. (2011)^47^ Taiwan	Cross‐sectional Post‐flood Three questionnaires via interview (not stated if in person) 3–6 months post‐flood	120; Mean age: 67 Females (60%) Education level of primary school or below (86.7%); Aged 55+ Convenience sampling	PTSD Symptom Scale Interview, Centre for Epidemiologic Studies Depression Scale, Disaster Severity Scale; Posttraumatic stress, depression	The proportion of participants with PTSD symptomology was 29.2% and among these people with PTSD symptomatology, 24 (68.6%, 24/35) had concurrent depression. Univariate analysis revealed that there were associations between PTSD symptomatology and gender, education level, personal injury, property damage, relocation, and self‐perceived health. Identified risk factors included the female gender, poorer self‐perceived health, relocation, family death, and depression. Our results support the finding that those people who were relocated had more severe symptoms of PTSD. Those relocated to temporary sheltered were likely to have experienced more property damage or personal injury, which were the main causes of PTSD symptoms. Even with these confounders controlled for, those relocated still reported higher levels of PTSD symptoms. Many of the individuals with PTSD were aboriginal. This rate of PTSD may result from the minority status of the aboriginal older people who are disadvantaged socioeconomically and receive fewer resources. In addition, the central belief in Taiwanese aboriginal culture is that ancestors’ spirits exist in the homeland and that it is necessary to protect and remain physically with these spirits. A belief that is held by many older adults who possess this traditional belief. Therefore, relocation and damage to the environment can conflict with their traditional beliefs and cause psychological difficulties.	The small sample size and time frame of data collection suggests the need for further research into this area. The results suggest that elderly people who are relocated require accompanying special psychological support services and the need for a special psychological rehabilitation program for those being relocated in the future, taking into account the culture and traditions of the individuals.
3	Ferraro (2008)^53^ USA	Longitudinal Post‐flood Five questionnaires in person 2–5 months post‐flood repeated for 2 years following	37; Mean age males (73.6, SD 4.67) Mean age females (74.79, SD 5.79) Females (62%) White (100%); Aged 60+; Convenience sampling	Geriatric Depression Scale (short form), Wechsler Adult Intelligence Scale—Revised, self‐rated health, medications taken, and questions about the flood; Depression	Depression scores were not affected across 3 years. Nor were there differences in gender for depression scores. The results were reported to seem consistent with the inoculation hypothesis, as all participants reported extensive exposure to floods and flooding, suggesting that prior experience with floods protected or insulated the individuals from strong emotional reaction to future events, as found by results on the depression scale.	Only used 37 individuals, who were all white and all high‐functioning; therefore, there may be other older adults who experienced these floods in alternate ways. This study examined few variables, therefore further measures would be helpful to better assess the impact of the flood.
4	Norris et al. (2002)^43^ Poland^a^	Cross‐sectional Post‐flood Two questionnaires via in person interview 6 months to 1‐year post‐flood	285, older adult sample *n* = 73; Mean age 67.2 (SD = 5.4) Female (57.5%) Education in years 11.5 (SD = 3.2); Aged 60+; Purposive sampling	PTSD Revised Civilian Mississippi Scale, Exposure measured on Likert scale; Posttraumatic stress	Severe distress was found in this population, with an average of eight PTSD criterion symptoms. Women exhibited more symptoms of PTSD than men. It is possible norms for mental health may be different, as in line with the literature. Poles have been found to score high on many measures of psychological distress compared to Americans. Polish culture encourages acceptance and expression of feelings and distress. The severity of flood event was an important predictor for PTSD symptoms. Individuals who experienced greater impact (such as injury, life threat, material losses, and social disruption) experienced greater rates of PTSD than individuals who did not. The more of these factors experienced, the greater the rate of PTSD. This study offered a contrasting view to the inoculation hypotheses, where prior experiences were expected to enhance coping abilities in older people. This study found older generations’ knowledge and skills acted as an obstacle to coping with new demands and rules, and were disproportionately affected by change.	Psychoeducational approaches to provide communities with information about normal versus severe or prolonged psychological reactions are needed to support victims, as there are many factors impacting PTSD symptom presentation.
5	Speis et al. (2019)^18^ Greece	Cross‐sectional Post‐flood and retrospective pre‐flood One questionnaire via interview (not stated if in person) 8 days post‐flood	78, older adult sample *n* = 16; Mean age not given. Age range 66–85 years Female (56.8%); Aged 65+; Purposive sampling	Self‐evaluation of perceived physical and mental health post‐disaster using 5‐point Likert scale; Mental health	Psychological health is maximized in the elder group. Older people tend to perceive the importance of floods as being greater than younger ones. Mortality in the urban area during the event was strongly associated with old age victims and the way they lost lives (inability to cope with flood waters, lack of rescue possibilities, dying helpless in their homes). Prior to the flood event, almost 60% described their psychological health as very good or good; however, after the flood event just under 16% rated themselves as very good or good, suggesting a decline in self‐reported psychological health. Females tended to report negative psychological health both prior to and post‐flood. Prior psychological problems were significantly associated with both physical and psychological problems following the flood.	Psychosocial needs are likely to differentiate between one disaster phase and the next, so needs need to be anticipated between stages. Impacts differentiate between gender, age, and occupational status, as well as other vulnerability factors. Recovery strategies need to include interventions and support, and these can be targeted to the most vulnerable groups. The primary concern of individuals in this study was the fear of reoccurrence, therefore it may be important for disaster policy to include public messages, and information about such possibility and disseminate information about reliable resources to access weather information.
6	Ti et al. (2016)^36^ Malaysia	Cross‐sectional Post‐flood One questionnaire via interview (not stated if in person) 8 weeks post‐flood	100; Mean age not given. Age range 60–80 years Female (53%) Married (75%); Aged 60+; Convenience sampling	Geriatric Depression Scale (Malay version), Trauma Screening Questionnaire (Malaysian version), Disaster Severity Scale; Depression, trauma	Depression was prevalent in 15% of participants. PTSD was prevalent in 17.8% of participants. Almost 50% of those experiencing depression in this study also had PTSD symptoms and those who had PTSD were five times more likely to also experiencing depression. 73.3% of the depressed group were surrounded by water, but houses were not damaged. Greater rates of house damage were found in the non‐depressed group. Being a woman, having PTSD, or a family history of psychiatric illness were risk factors for depression.	Interventions must consider mental, physical, social, economic, and spiritual/religious support, and group support may be beneficial as family plays a key role. The questionnaire was limited in assessing important factors related to post‐disasters such as bereavement, social or physical support, or number of days evacuated which could provide vital information.
7	Tyler and Hoyt (2000)^37^ USA	Longitudinal Pre‐ and post‐flood Four questionnaires via in person interview 1 year pre‐flood, 60 days post‐peak of flood	651; Mean age not given. Age range 55–70+ Female (69%); Aged 55+; Convenience sampling	Centre for Epidemiologic Studies Depression Scale, Flood exposure questionnaire, Social Provisions Scale; Depression, acute stress	Prior level of depression was a significant predictor for current depressive levels. This is a clear vulnerability factor. Social support was important for alleviating the effects of depressive symptoms. Individuals exposed to flooding were more likely to experience depressive symptoms in the younger of older adults (aged 55–69 years). When flood exposure was high and social support was low, depression levels increased. Individuals who had access to social support when needed were better able to cope. Individuals with little or no social support may have a more difficult time dealing with life changes, and are more vulnerable to increases in depression.	Future research needed to replicate findings among similar age groups. Little research has examined how stress and depression are moderated by social support and how this might operate differently for distinct age groups. Testing for age interactions allows for evaluation of whether the effects of an acute stressor on depression may vary by level of social support for distinct age groups.
8	Wind et al. (2011)^60^ UK	Cross‐sectional Post‐flood Five questionnaires via in person interview 11 months post‐flood	231, older adult sample *n* = 108; Characteristics of whole sample: Mean age not given. Female (60.8%) Religious (95.9%) Married (35.8%); Aged 65+; Purposive sampling	Hopkins Symptom Checklist, PTSD Checklist Civilian Version, Social Support Scale, Using 5‐point Likert scales the following were measures: Displacement, Appraisal Processes, Coping Intensity; Depression, anxiety, PTSD.	Cognitive social capital^b^ consistently related to lower mental health problems and structural social capital^c^ was associated with more anxiety but not PTSD or depression. Feelings of cohesiveness (cognitive social capital) may protect against depressive illness, but participation in social structures (structural social capital) may be associated with an excess of anxiety disorders. Perceptions of higher trust and mutual help (e.g. cognitive social capital) decreased the negative relationship between coping intensity^d^ and mental health outcomes.	Individual‐oriented stress‐reducing interventions that use appraisal processes, social support, and coping as starting points could be more effective by accounting for subjective experience of the social context in terms of trust and feelings of mutual support and reciprocity in a community. Affected people may benefit from combining individual stress‐reducing interventions with psychosocial interventions that foster cognitive social capital.
9	Wind et al. (2021)^54^ UK	Cross‐sectional Post‐flood Five questionnaires via in person interview 1 year post‐flood	231, older adult sample *n* = 138; Characteristics of whole sample: Mean age not given. Female (61%) Religious (85.3%) Married (35.9%); Aged 65+; Purposive sampling	Hopkins Symptom Checklist, Shortened and adapted Social Capital Assessment Tool, Using 5‐point Likert scales the following were measured: Collective Efficiency Scale, Residential Stability, Disaster Property Loss, Primary Appraisal, Coping Effort; Depression	Consistent with the existing literature, being personally affected by the floods was associated with significantly higher levels of PTSD symptoms on all three assessed domains. Community social capital is related to post‐disaster depression problems. In communities with high social capital, there was less associated suffering from symptoms of depression. Older people received less social support and appeared more isolated. Perceiving the disaster as less traumatic after a year was related to more feelings of depression.	Independent measures of community variables would be beneficial in future studies. Older adults need to be targeted for individual psychosocial interventions to limit their dependency on community support. This study was cross‐sectional and so did not allow for causal inferences. Longitudinal studies should be conducted to address this gap.
10	Wind and Komproe (2012)^61^ UK	Cross‐sectional Post‐flood Eight questionnaires via in person interview 1‐year post‐flood	231, older adult sample *n* = 108 Mean age not given. Characteristics of whole sample: Female (60.8%) Religious (94.9%) Married (35.8%); Aged 65+; Purposive sampling	PTSD Checklist Civilian Version, Shortened and adapted Social Capital Assessment Tool, Using 5‐point Likert scales the following were measured: Collective Efficiency Scale, Residential Stability, Disaster Property Loss, Primary Appraisal, Coping Effort; PTSD	Social context acted as a protective factor against disaster‐related distress whereby higher social capital equated to less posttraumatic stress. Higher social capital is linked to the preservation of individual psychosocial resources, which in turn is associated with lower levels of posttraumatic stress. This suggests individuals with high social capital suffer less from disaster‐related distress.	Research into other types of mental health problems such as depression and substance abuse are required, since they might not share a common pattern of association with social capital. Community interventions that foster social capital over traditionally individual psychological interventions may promote positive outcomes effectively with fewer resources, decreasing the need for individual psychological interventions.

^a^ Also looked at other natural disasters in the United States and Mexico.

^b^ Cognitive social capital refers to: trust, social harmony, perceived fairness, and sense of belonging.

^c^ Structural social capital refers to: membership of groups, involvement in citizenship activities, and social support from the community.^86^

^d^ Coping intensity refers to: the degree to which a variety of coping strategies were employed.^60^

Abbreviation: PTSD, posttraumatic stress disorder.

### Study characteristics

Studies most commonly reported cross‐sectional designs (70%), in high‐income countries (100%), with surveys completed between 8 days and 1 year following a flood event. Two studies described their design as longitudinal (20%), and one cohort design collected data pre‐ and post‐flood (10%). Geographically, the studies spanned four continents: five in Europe, two in the United States, two in East Asia, and one in Australia. Studies spanned three decades, with three being published between 2000 and 2010, six published between 2010 and 2020, and one published between 2020 and 2023.

Studies varied in their definition and inclusion of older adults. Five reported samples of adults aged 18+, but age was separated to compare groups; four of the studies discussed older adults as aged 65+, one termed older adults as aged 60+, as this was seen to align most appropriately to a later life stage in the relevant country (Poland). Two studies included older participants only, using a sample of individuals aged 55+; again this was deemed by the researchers to meet inclusion criteria as the authors argued how this age had appropriately reflected adults in a later stage in these populations of investigation (Taiwan and United States). Three studies investigated older adults only, using a sample of individuals aged 60+.

### Quality of studies

Overall, the studies in this review were rated weak to moderate quality. Out of the 10 articles included, 80% were found to be weak, 20% (*n* = 2) were rated as being of moderate quality,^36^, ^37^ and no studies were found to be of strong quality (Table 2). It should, however, be noted that due to the nature of disaster‐related studies, some of the quality criteria cited in the tool are impossible to meet (e.g., randomization and control groups).^38^ These were common issues contributing to lower quality ratings. For example, all studies scored as weak on blinding to the flood event. Similarly, due to the likelihood of this population being relocated following a flood event, and all of the sampling methods involving convenience or purposive sampling methods of flood‐damaged properties, it is possible many of the target sample were not present for participation in studies, leading to most of this category on the quality assessment scale being deemed weak due to sampling bias. It is important to recognize the probable issues in post‐disaster areas in terms of withdrawal, and this has implications for an overall evaluation of the research quality, as well as highlighting issues that future researchers may wish to consider when designing future studies on this topic. The main difference between the two studies categorized as moderate quality in this review, compared to the remaining weak studies, is that they reported minimal withdrawal/drop out levels^36^, ^37^ in contrast to this being a limitation of most of the other studies. These two studies varied in design, sample size, and country of research, making it difficult to determine the approach that contributed to obtaining greater response rates, leading to a higher quality study.

**TABLE 2:** Quality assessments.

Study	Criteria						Overall rating
	Selection bias	Study design	Confounders	Blinding	Data collection methods	Withdrawals and drop‐outs	
Bei et al.^46^	Weak	Moderate	Strong	Weak	Strong	Moderate	Weak
Chen et al.^47^	Moderate	Weak	Strong	Weak	Strong	Moderate	Weak
Ferraro^53^	Weak	Moderate	Strong	Weak	Weak	Strong	Weak
Norris et al.^43^	Moderate	Weak	Strong	Weak	Strong	Moderate	Weak
Speis et al.^18^	Moderate	Weak	Strong	Weak	Weak	Moderate	Weak
Ti et al.^36^	Moderate	Moderate	Strong	Weak	Moderate	Strong	Moderate
Tyler and Hoyt^37^	Moderate	Moderate	Strong	Weak	Strong	Strong	Moderate
Wind et al.^60^	Weak	Moderate	Strong	Weak	Strong	Moderate	Weak
Wind et al.^54^	Weak	Moderate	Strong	Weak	Strong	Moderate	Weak
Wind and Komproe^61^	Weak	Moderate	Strong	Weak	Strong	Moderate	Weak

*Note*: Combined scores from two researchers (S.L. and L.E.) calculated using the Quality Assessment Tool for Quantitative Studies.

### Overview of evidence into the impact of flooding on the mental health of older adults

All studies described the exposure as a major flood event or disaster, and most were described as the worst for many years, causing extensive damage and loss. The floods resulted in significant material damage, many individuals were killed, injured, missing, or they were evacuated due to property flooding or concerns about safety. There were extensive financial losses and extortionate clean‐up costs. Specific details regarding the scale and damage from each flood are outlined in Table S2.

Most studies explored the impact of flooding on depression (70%), posttraumatic stress (60%), and/or anxiety (20%). Over half of the studies considered other impacts and protective factors such as physical health impacts, appraisals of the event, social support, and ability to cope. A variety of measures were used to assess mental health impacts, and the outcomes described below are based on these heterogenous measures. Posttraumatic stress was assessed using four different measures, depression was assessed using three distinct measures, and anxiety was assessed using two differing measures.

### Posttraumatic stress

Four measures were used to assess posttraumatic symptoms in older adults following flooding: The Impact of Events Scale—Revised (IES‐R), PTSD Symptom Scale (PSS), PTSD Revised Civilian Mississippi Scale (PCMS), and the Trauma Screening Questionnaire (TSQ). The IES‐R investigates a subjective response to a specific traumatic event in the older adult population. It has been found to be effective in use with both healthy and frail older adults exposed to any specific traumatic event.^39^ It has been translated into multiple languages for use globally. Despite being reported as a helpful tool for screening PSTD symptoms such as intrusions (thoughts, nightmares, etc.), avoidance, and hyperarousal,^39^, ^40^ it is difficult to find evidence that supports its use on older adult populations outside of the veteran population, suggesting a need for reliability and validity checks in the general older adult population. As this is not a diagnostic tool for PTSD, there are discrepancies with how it links to the DSM criteria for this disorder, making comparisons difficult.^40^ The PSS was used in one study and is a widely used interview measure of PTSD that is based off the DSM‐5. It was found to have excellent psychometric properties,^41^ but again its psychometric properties in nonveteran populations are not well known.^42^ The PCMS was used in four studies in this review to measure PTSD symptoms. This scale has excellent cross‐language psychometrics^43^ and was found to be a reliable and valid tool for the assessment of PTSD symptoms, specifically in veterans.^44^ The TSQ was used in one study included in this review and was translated into Malaysian, showing cross‐language use. It has good psychometric properties that appear consistent across countries,^45^ suggesting this could be a useful tool for global studies. The TSQ was derived from the PSS, using items of re‐experiencing and arousal symptoms to determine PTSD symptoms. Similar to the other measures discussed, there are few papers reporting the usefulness of this tool in terms of the general older adult population. It is clear from reviewing the tools used that there is a need for a consistent, globally appropriate tool that is validated in the general older adult population for assessing PTSD symptomology following a natural disaster.

Individuals personally affected by flooding reported greater rates of posttraumatic stress symptoms on these outcome measures.^46^ As might be expected, posttraumatic stress symptoms were particularly elevated when the experiences of flooding involved significant traumatic and disruptive life events including risk of life, injury, material losses, relocation, and social disruption.^43^ Multiple studies reported greater rates of posttraumatic stress in women compared to men.^43^, ^47^ Posttraumatic symptoms were associated with relocation, poor self‐perceived health, and experiencing a loss of a family member.^47^ An association was found between those who received governmental support and higher rates of posttraumatic stress,^46^ and the authors recognized that this relationship was likely due to government support being offered more to those individuals who had been most severely affected. One study in Taiwan^47^ reported that within their sample, most of the individuals who were experiencing posttraumatic stress symptoms were aboriginal, a group that was known to be more socioeconomically deprived and less resourced compared to other ethnic groups in Taiwan, and as seen elsewhere, lower socioeconomic status is associated with increased mental health symptomatology post‐flooding. In addition, the authors described their findings in relation to cultural norms and discussed an important belief held in Taiwanese aboriginal culture where ancestors’ spirits exist in the homeland, and that current generations must protect and be physically present with the land and their ancestors. As such, for this group of aboriginal Taiwanese individuals, it is possible that both damage to the environment and the need to relocate could have a significant meaning in relation to their belief system and this in turn may hold explanatory power for why this group may experience increased rates of psychological distress, such as posttraumatic stress symptoms.

### Depression

Three measures were used to identify depressive symptoms in older adults following flooding: Centre for Epidemiologic Studies Depression Scale (CES‐D), Geriatric Depression Scale (GDS), and Hopkins Symptom Checklist (HSC). The CES‐D has been validated in many populations, including the elderly, and was found to be easy to read and understand, and quick to complete.^48^ However, some evidence suggests that the cut‐off score produces a high percentage of false positives, therefore many studies use varying cut‐off scores, to account for sensitivity and specificity.^49^ It can be used with a variety of populations due to the many translated versions available, the third‐grade reading level, and the side use as a research tool; however, as it does not measure all symptoms of depression (e.g., suicidal ideation), it may not address intended aims.^49^ The GDS was developed specifically for the older adult population in the affective and cognitive domains. The GDS does not consider some core symptoms of depression such as suicidality, appetite, sleep, or energy levels.^49^, ^50^ It has been used across cultures due to its many language translations and is written to a grade 4 reading level, making it largely accessible.^49^ The HSC similarly has been validated in the older adult population and is sensitive to pain, distress, and impairment in this population. It was found to have a low rate of false positives and high sensitivity for depression.^51^ It has been translated into nine European languages with appropriate cultural checks for use across European countries^52^; however, it is not clear that it has been translated for use across continents making it less accessible or available for global studies. Clearly there are various tools available to screen for depressive symptoms in older adult populations; however, there is limited evidence to suggest which tool is best equipped to measure post‐disaster depression in the elderly population. More research is needed into the quality and applicability of these tools across cultures following disasters.

Older adults were found to experience high rates of depression on these outcome measures following flood exposure. Individuals who experienced depression prior to the flood experienced higher levels of depression post‐flood.^37^ Other risk factors included being a woman, having posttraumatic stress symptoms (as described above), and/or a family history of psychiatric illness.^36^ Prior experience of floods acted as a protective factor against higher scores on the depression scale,^53^ and social support was found to be important for alleviating symptoms of depression.^54^ There were associations between posttraumatic stress and depression, with almost half of those experiencing depression also exhibiting posttraumatic stress symptoms, and those with posttraumatic stress symptoms found to be five times more likely to experience depression.^36^

### Anxiety

Anxiety was measured using two different measures in the papers included in this review: The HSC as discussed earlier has been validated in older adult populations and translated into multiple languages in Europe.^52^ However, when assessing anxiety symptoms, it was found to be difficult to identify an optimal cut‐off point for anxiety symptoms to obtain a good degree of sensitivity and predictability.^55^ Furthermore, there are limited validation studies that have been conducted in non‐Westernized populations, highlighting a need for this for use in global studies.^55^ The Geriatric Anxiety Inventory (GAI) was used in one study included in this review and is effective in accounting anxiety in the older adult population.^56^ It is used to assess anxiety symptoms in general, so is not used as a diagnostic tool^57^ and has been translated into multiple different languages, where validation research has been investigated across countries with comparable scores present.^58^, ^59^ There are very few studies that report the effectiveness of this tool for measuring anxiety post‐disaster and so investigation is warranted to ensure it can be effectively used in a study looking at the impact of a disaster on anxiety symptoms.

Older adults who were directly affected by flooding experienced increased rates of anxiety as found on the outcome measures used, particularly when personal loss and community destruction was evident.^46^ It was also found that higher levels of anxiety were associated with structural social capital, suggesting that feelings of anxiety can be exacerbated by close social structures.^60^

### General mental health

Many studies reported a significant increase in psychological distress in the older adult population following exposure to flooding, linked to both mood and satisfaction with life.^18^, ^46^ Worse mental health outcomes were found when individuals were evacuated, had their routine disrupted, experienced financial loss, or received limited or no social support.^46^ Individuals who experienced mental health difficulties prior to the flood events experienced greater rates of physical and psychological problems following the flood.^18^ Multiple studies recognized being woman as a risk factor for increased psychological difficulties.^18^, ^43^

### Protective factor—Prior experience of flooding

Two studies discussed Eysenck's 1983 inoculation hypothesis, which posits that prior experience with natural disasters acts as a protective factor from distress and strong emotional reactions if this same disaster occurs again. One study supported this hypothesis, evidencing a link between having previous experience of flooding being associated with lower rates of depression.^53^ It is worth noting that this finding was not universal, as one study^43^ reported that older generations were hindered by their knowledge and skills as they became less able to cope with new demands and were disproportionately affected by change, despite prior experiences. Furthermore, both studies reporting their findings on the inoculation hypothesis were of weak quality, a finding that indicates the need for more, higher quality studies to ensure accurate understanding of how prior experiences of flooding affect mental health after a flood.

### Protective factor—Social support post‐disaster

An important focus for some of the papers was to investigate any factors that had the potential to mitigate poor mental health outcomes. The most investigated factors were community cohesion and social support using a variety of validated evaluation tools.^37^, ^54^, ^60^, ^61^ Four studies noted that individuals who received greater social support, such as psychological aid, group support, or access to transportation through family vehicles, experienced lower levels of depression and posttraumatic stress symptoms, as reported by the measures used. Similarly, having a strong sense of community cohesion and support, such as positive relationships with the neighborhood, was associated with better mental health outcomes and lower reported depression following flooding. These findings emphasize the importance of social support as a protective factor against poor mental health outcomes following disasters. This is similar to the wider literature that reports social support mediates between disaster distress and mental health outcomes.^62^ Despite the general finding that social support could offer a useful tool to reduce psychological distress, the tools and measures used across these studies varied, which suggests that there could be scope for global studies aiming to use a reliable global assessment of social support following disasters. Without this, as seen in this review, it is challenging to compare countries with accuracy. Furthermore, those studies assessing social support were largely cross‐sectional, preventing conclusions being drawn regarding the directionality of relationships between social support and distress. Research may benefit from the inclusion of qualitative data and mixed methods designs to better understand individuals’ experiences, particularly across cultures.^63^

### Intervention suggestions

Most studies discussed how outcomes could be improved for older adults who experience floods. Of particular importance, the strengthening of social support networks at both individual and community levels appeared to play a significant role in promoting resilience and aiding recovery. Providing early‐stage social support interventions post‐flood was hypothesized to reduce the risk of developing mental health issues such as depression, anxiety, and posttraumatic stress. Interventions to strengthen community connections and resources would provide a supportive environment for older adults, which could indirectly reduce individual posttraumatic stress symptoms and promote mental health. This adds to existing claims that interventions must incorporate social cohesion, which is associated with improved resilience and decreased symptoms of psychological distress.^64^, ^65^

This review also highlights the potential importance of early interventions post‐flood, in line with the general literature where early interventions such as psychological first aid, psychological debriefing, or crisis counselling can reduce distress, with emotional support having the potential to reduce the likelihood of an individual developing a mental health disorder. However, such interventions have not been empirically tested, and more research must examine the extent of help or even harm they may cause post‐disaster before informing practice.^66^ However, it may be helpful to identify those most at risk of poorer mental health to target early interventions. Such early interventions could incorporate both individual coping strategies and community‐level support to address the multifaceted nature of psychological challenges after flooding, to promote safety, improve self‐efficacy, and encourage social connection, to aid recovery from trauma.^67^ These are all essential targets for future research in older adult populations after climate disasters.

## DISCUSSION

This timely scoping review assessed the state of the quantitative evidence for the impact of flooding likely related to climate change on the mental health of older adults. With only 10 articles meeting the inclusion criteria, it is clear that research into specific climate change events (as defined by the IPCC and World Health Organization) and older adults is scarce compared to other age groups.^26^, ^27^ The lack of literature and the lack of high‐quality studies are particularly notable considering that older people are known to have more physical and mental health vulnerabilities to the effects of flooding. The literature on older adults predominantly focused on the physical health impacts, indicating the lack of emphasis of the equally important effects on mental health and well‐being, and a potential bias in research priorities. This review clearly shows both that the current evidence base lacks size and robustness and that more research is necessary, particularly since in the coming decades, floods will increase in frequency, severity, and duration,^68^ alongside the aging of the global population.^7^

Older adults were defined as individuals aged over 55 years in this study, to attempt to capture the life stage of being an older adult. It is suggested that categorizing older adults should not be defined strictly by a specific age, as individuals biologically age at differing rates, and factors such as mobility, frailty, and required care may be more important considerations.^69^ Another element relevant to determining this life stage is retirement, something that varies across country and with socioeconomic status. The choice of age bracket used in this review is both a strength in terms of global inclusivity and a limitation in terms of variation across countries, which affects fair comparability. For example, individuals who are retired generally experience higher levels of psychological distress than those in paid work,^70^ possibly due to biological changes or illnesses that occur with aging, and societal transitions such as loss of roles and isolation.^71^ These factors might be relevant to how older adults experience distress post‐disaster too, but this was not measured specifically. Relatedly, mental health inequalities in older adults (as in other groups) are associated with lower economic status and/or lesser education,^72^ but only one article reviewed considered socioeconomic factors contributing to differences in mental health outcomes, so the importance in post‐disaster outcomes remains an unclear factor. Another key factor that was under‐reported was the contribution of disability, particularly mobility impairments and dependence on regular treatments (e.g., symptom management, care, and medication), to mental health post‐flood. Such factors will impede recovery from disasters, and barriers to accessing care needs will lead to increased morbidity and even mortality.^73^ Since physical and mental health are reciprocal relationships, more complex study designs may be required to better understand how mental and physical health outcomes interact post‐flooding.^74^ Future research may benefit from including these multiple factors and improvements in definitions of older adults across countries.

Across the studies reviewed, the variety of measures assessing mental health and social support indicates a pressing need to understand and improve the quality of outcome tools globally. Developing more universal and cross‐culturally appropriate tools to assess mental health post‐disasters would allow for more accurate comparisons of impact, so that world leaders can better understand how to support individuals following disasters, and where the most help might be needed. Obviously, universal measures have their own challenges, and developing and testing such tools will require sensitive consideration of cultural differences to ensure they are reliable and valid for the intended population.^75^ This in turn will support the development of culturally appropriate interventions to enhance recovery and support for individuals and communities following natural disasters.

Overall, flooding‐related climate change has a clear impact on the mental health of older adults. Effects include high levels of posttraumatic stress, depression, anxiety symptoms, and overall psychological distress, which have been evidenced for decades. This aligns with the growing evidence base more broadly showing the detrimental and devastating effects of climate change and related natural disasters on mental health.^2^, ^16^, ^17^, ^27^ This is presented with the caveat that more high‐quality studies are required to ensure this conclusion is robust, and to give a clearer sense of the sizes of effects and what factors are important. Furthermore, future studies should investigate other aspects of mental health likely to be impacted by climate‐related events, such as suicidality, insomnia, substance abuse, behavioral disturbances, and cognitive difficulties.^15^

Studies identified multiple factors increasing risk of mental illness post‐flood, including experiencing mental health difficulties prior to flooding, a history of mental health difficulties in the family, being female, and being from a lower socioeconomic class or lower education level. These findings were found in both weak and moderate quality studies, suggesting this may be a more reliable finding, and are also consistent with systematic reviews into risk factors for mental health in older adults generally, which have shown that those who had prior mental health difficulties, are female, or have lower education or lower socioeconomic status are at higher risk of mental health difficulties.^76^, ^77^ A major issue with the research scoped in this area is the lack of studies in LMICs, where the risks attached to flooding are already higher.

Social support appeared to be an important protective factor for mental health, as noted in earlier literature.^78^ Those reporting more social support reported lower rates of depression following flooding. This is in line with the literature that discusses the importance of social support in the aftermath of natural disasters more broadly.^17^ Relatedly, the review suggests the possible value of early interventions in older adults post‐flood, although much more research is required. In general, early posttraumatic interventions have been posited to lead to helpful reappraisals of events, more adaptive reactions, and less posttraumatic symptomology,^79^ but evidence here lacks robustness,^66^ and it would be too soon to claim this approach would be broadly applicable following large‐scale disasters. Clearly we need urgent research to develop appropriate early responses that can reliably promote psychological health in older adults post‐disaster. Relevant designs would include large scale longitudinal or cohort studies monitoring mental health outcomes over time.

### Study characteristics including methodological approaches and quality assessment

Despite spanning three decades, there were little differences in the findings across the articles reviewed. The increasing number of studies from 2010 onward perhaps reflects more frequent flooding^68^ and growing awareness of this as an issue. Future decades will sadly bring increasing occasions for further research into this area.

The lack of research conducted in LMICs severely limits our understanding of older adult mental health in the context of flooding globally. Considering that LMICs have experienced some of the most devastating climate‐related floods (e.g., Pakistan in 2022), this omission is a significant risk to the degree to which the scoped literature can be said to reliably reflect the experiences of older adults from a global perspective. Higher income countries such as the United States and across Europe tend to have greater resources available to respond quickly and at scale to disasters compared to LMICs,^68^ and there are significant cultural differences in community cohesion and attitudes toward aging in different parts of the world. Other cultural issues include the fact that indigenous populations are often marginalized, and hold strong relationships and identity within their environment which may make flooding and associated relocation particularly painful.^47^, ^80^ We caution against extrapolating findings from this review to a global population and urge researchers to turn their attention to neglected regions of the globe.

Sampling approaches also limit the conclusions that can be drawn, with participants selected through convenience or purposive sampling, and significant variation between sample sizes reported. This again limits how well the existing literature can offer a general overview of older people globally. The reliance upon cross‐sectional survey design is helpful in pointing toward potentially important factors and findings, and is understandable considering the nature of the phenomenon being investigated. However, a lack of pre‐flood data and control groups prevents drawing conclusions regarding causality or longer term mental health outcomes and recovery. The variability in timing of data collection (ranging from 2 months to 1 year post‐flood) gives some sense that impacts can be medium term, but there is a scarcity of studies investigating long‐term effects of flooding.^27^ Timing variability again creates challenges when attempting to compare studies or pool results. Again, longitudinal and cohort studies with repeated measure designs would improve our understanding and help to investigate potential mediators and moderators such as social support pre‐flood.

It is worth noting the quality appraisal of studies, where all but two were rated low quality. As stated earlier, the chosen quality assessment tools consider the risk of bias in cohort studies to be great, and do not account for the fact that natural events such as flooding cannot be adequately blinded or controlled in line with the gold standard of health research.^38^ Selection bias was also high because of a reliance on purposive or convenience sampling, which is often necessary when attempting to collect data in a situation where there may be severe structural limitations from flooding damage, and significant post‐disaster relocation of individuals and communities, leading to smaller and fewer samples. Future research should consider how to manage this, particularly as repeated flooding may lead to longer term displacement and relocation, which itself has significant relationships with poor mental health.^43^, ^47^ This study purposely did not exclude studies on quality, instead aiming for a comprehensive picture of existing literature, but the implications are clear in terms of the need for greater quality in future research.

### Strengths, limitations, and future recommendations

The broad and inclusive review strategy is a strength of this, and other scoping reviews,^81^ particularly for an emerging area of interest. With few studies delving into the impacts of specific types of climate events on mental health and older adults, the flexible and exploratory nature of this approach was appropriate, and, to date, there is insufficient high‐quality evidence to warrant a different type of synthesis such as meta‐analysis on this topic.

No papers were included reporting on countries most vulnerable to the effects of climate change in line with the Notre Dame‐Global Adaption Initiate Index, the top five of which are all located in the continent of Africa (Chad, Central African Republic, Eritrea, Democratic Republic of Congo, and Guinea‐Bissau). The restriction placed on including studies available in the English language may well have contributed to this limitation but was unavoidable due to lack of resources for translation. Another limitation is that the scope of this review meant it was limited to including quantitative methodologies only, and by excluding qualitative studies, it is possible that important findings, particularly relating to minoritized voices who may bear disproportionate climate burdens, were lacking. For example, another recent review discusses the impacts of floods in Pakistan.^82^ We also point the reader to the recently published meta‐ethnographic evaluation of global qualitative literature of mental health impacts of climate change more broadly upon older adults.^83^

As such, this review is limited in what conclusions can be drawn regarding the mental health of older adults in LMICs with less developed infrastructure such as drainage and flood protection, and where floods likely cause more long‐lasting and devastating damage, and where there are more inequalities in social systems and resources to support the marginalized, and less developed insurance markets.^84^ In addition, the stigma of mental health difficulties is still seen as a significant challenge in some LMICs^46^ and therefore accessing appropriate support or participating in research relating to mental health may not be as accessible to individuals living here. More needs to be done in considering engagement and accessibility with communities that are harder to reach, using culturally appropriate approaches.

The search terms were developed to attempt to include studies where disasters (including flooding) could be seen as likely related to climate change. Based on the scientific consensus of climate change increasing the risk of floods, it was agreed that significant flood events since 2000 were at least somewhat likely to be related to climate change. The authors acknowledge that some flood events could have occurred without climate change. They also argue that the impact of the flood on the mental health of older adults is still relevant to climate change because floods due to natural disasters have very similar outcomes regardless of how important climate change was as a contributing factor. This argument is aligned with previous reviews, and the recognition that determining a direct link between rising global temperatures and any one specific flood event is impossible, and needs to be seen from the perspective of probabilities (i.e., global warming increases the likelihood that an extreme weather event occurs).^24^, ^26^, ^27^

The decision to limit the inclusion date to the year 2000 onward means that some studies may have been missed for inclusion in this review. The justification is that this date reflects growing awareness of climate change and mental health impacts, and again aligns with previous approaches.^8^, ^30^ A further limitation of this study was that 3 of the 10 articles included in this review used data collected from the same sample, restricting the breadth of findings and perspectives reported.

This review summarizes the state of the current evidence base into the impact of flooding on the psychological health of older adults. In doing so, it has highlighted some important directions for future research. First and foremost, it indicates that there is a high demand for more up‐to‐date research into the psychological health of older adults following flooding, as these events are increasing in frequency and severity worldwide. There is a need for a wider variety of mental health conditions to be considered, in addition to the ones discussed in the papers included in this review, as difficulties such as suicidality, substance abuse, and guilt are also recognized as potential experiences for this population following disasters.^17^

### Future directions

Future research should employ more robust research methodologies and recruitment techniques to engage older adults, particularly those who have been relocated, and reduce dropout rates. Far more larger scale work is required with the global majority living in LMICs, particularly those with high flood risks. Longitudinal and cohort study designs could be developed by collecting data in areas known to be at high flood risk before flooding, and to collect data at several time points post‐flood to examine longer term impacts. Collected data need to be more varied and include aspects such as disability, frailty, mobility, retirement status, access to resources (e.g., warning messages about impending disasters), and socioeconomic status. Work should also be done on developing and validating appropriate cross‐cultural measures for older adults globally, which recognize that psychological health is perceived differently across countries,^82^ as is the frequency, intensity, and type of disaster experienced. A future review could explore what tools are available and make recommendations for researchers.

## CONCLUSION

There is a small but growing literature that indicates that climate‐related flooding has important impacts on the mental health of older adults. Despite limitations in the study designs, there appear to be consistent associations between flooding and increased rates of depression and PTSD among older adults, aligning with the existing literature on climate change's adverse effects on mental health more broadly. Various factors associated with mental health outcomes were identified, in particular that a lack of social support predicts worse mental health outcomes. Researchers in this area have claimed that early interventions post‐flood can promote mental health resilience and recovery. However, reliable intervention research is lacking. Future researchers should consider testing whether enhancing community and individual resilience through building social networks could improve outcomes in this group. More research is needed in LMICs and vulnerable regions most affected by climate change.

## AUTHOR CONTRIBUTIONS

**Sarah Law**: Conceptualization; methodology; formal analysis; writing—original draft; writing—review and editing; visualization. **Temenuzhka Marinova**: Conceptualization; methodology. **Lillie Ewins**: Formal analysis. **Elizabeth Marks**: Conceptualization; methodology; writing—review and editing; supervision.

## CONFLICT OF INTEREST STATEMENT

The authors declare no conflicts of interest.

## PEER REVIEW

The peer review history for this article is available at https://publons.com/publon/10.1111/nyas.15356.

## Supporting information



Supporting Information

## Data Availability

Data sharing is not applicable to this article as no datasets were generated or analyzed during the current study.
