# Supplementary material for: Understanding the psychological impact of flooding on older adults: A scoping review
Source: Ann N Y Acad Sci. 2025 May 14;1548(1):99–115. doi: 10.1111/nyas.15356 (PMC12220290; doi:10.1111/nyas.15356)
Supplement: Supplementary file 1 — Supporting Information [file NYAS-1548-99-s001.docx]

# **Supporting Material**

| **Supporting Information S1:** *Search Strings for Each Database* |
| --- |
| **APA PsycNet, including PsycExtra**  ((((KEYWORDS: ("mental health")) OR (KEYWORDS: ("mental illness")) OR (KEYWORDS: ("mental disorder")) OR (KEYWORDS: ("psychiatric disorder")) OR (KEYWORDS: (anxi*)) OR (KEYWORDS: (panic)) OR (KEYWORDS: (depress*)) OR (KEYWORDS: (distress)) OR (KEYWORDS: ("post traumatic")) OR (KEYWORDS: (PTSD)) OR (KEYWORDS: ("bipolar disorder")) OR (KEYWORDS: ("mood disorder")) OR (KEYWORDS: (schizophren*)) OR (KEYWORDS: (psychosis)) OR (KEYWORDS: (suicid*)) OR (KEYWORDS: (emotion*)) OR (KEYWORDS: ("eco anxiety")) OR (KEYWORDS: (ecoanxiety)) OR (KEYWORDS: (grief)) OR (KEYWORDS: (solastalgia)) OR (KEYWORDS: (worry*)) OR (KEYWORDS: (worries)) OR (KEYWORDS: (fear))))) AND ((((KEYWORDS: ("climate change")) OR (KEYWORDS: (greenhouse)) OR (KEYWORDS: ("global warming")) OR ((KEYWORDS: (temperature*)) AND ((KEYWORDS: (rise*)) OR (KEYWORDS: (rising)))) OR (KEYWORDS: ("sea level")) OR (KEYWORDS: ("heat wave")) OR ((KEYWORDS: (extreme)) NEAR/1 ((KEYWORDS: (temperature*)) OR (KEYWORDS: (weather*)) OR (KEYWORDS: (heat)) OR (KEYWORDS: (cold) OR (KEYWORDS: (events)))) OR (KEYWORDS: (flood*)) OR (KEYWORDS: (drought*)) OR (KEYWORDS: ("forest fire*")) OR (KEYWORDS: (wildfire)) OR (KEYWORDS: (bushfire)) OR (KEYWORDS: ("natural disaster*")) OR (KEYWORDS: (storm*)) OR (KEYWORDS: (typhoon*)) OR (KEYWORDS: (hurricane*)) OR (KEYWORDS: (cyclone*)) OR (KEYWORDS: ("vector borne disease")) OR (KEYWORDS: (desertification)) OR (KEYWORDS: (deglaciation)) OR (KEYWORDS: (deforestation)) OR (KEYWORDS: (ecomigration)) OR ((KEYWORDS: (climate NEAR/6 adaptation))) OR ((KEYWORDS: (climate NEAR/6 mitigation))) OR ((KEYWORDS: (climate NEAR/6 resilience)))))) AND ((((KEYWORDS: (geriatric)) OR (KEYWORDS: (senior*)) OR (KEYWORDS: (elder*)) OR (KEYWORDS: (aged)) OR (KEYWORDS: (ageing)) OR (((KEYWORDS: (old)) OR (KEYWORDS: (older))) AND ((KEYWORDS: (people)) OR (KEYWORDS: (adult*))))))  Filters: English, years 2000 - 2024  APA PsycNet, including PsycExtra: the same search string as above in the Abstract Field  **PubMed**  #1 (mental[Title/Abstract] AND (health[Title/Abstract] OR illness[Title/Abstract] OR disorder[Title/Abstract])) OR "psychiatric disorder"[Title/Abstract] OR "mood disorder"[Title/Abstract] OR anxi*[Title/Abstract] OR panic[Title/Abstract] OR depress*[Title/Abstract] OR distress[Title/Abstract] OR "post traumatic"[Title/Abstract] OR "post-traumatic"[Title/Abstract] OR PTSD[Title/Abstract] OR "bipolar disorder"[Title/Abstract] OR schizophren*[Title/Abstract] OR psychosis[Title/Abstract] OR suicid*[Title/Abstract] OR emotion*[Title/Abstract] OR ecoanxiety[Title/Abstract] OR grief[Title/Abstract] OR solastalgia[Title/Abstract] OR worry*[Title/Abstract] OR worries[Title/Abstract] OR fear[Title/Abstract] #2 "climate change"[Title/Abstract] OR greenhouse[Title/Abstract] OR "global warming"[Title/Abstract] OR (temperature[Title/Abstract] AND (rise*[Title/Abstract] OR rising[Title/Abstract])) OR "sea level"[Title/Abstract] OR "heat wave"[Title/Abstract] OR heatwave*[Title/Abstract] OR "extreme temperature*"[Title/Abstract] OR "extreme weather*"[Title/Abstract] OR "extreme heat"[Title/Abstract] OR "extreme cold"[Title/Abstract] OR "extreme events"[Title/Abstract] OR flood*[Title/Abstract] OR drought*[Title/Abstract] OR "forest fire*"[Title/Abstract] OR wildfire*[Title/Abstract] OR bushfire*[Title/Abstract] OR "natural disaster*"[Title/Abstract] OR storm*[Title/Abstract] OR typhoon*[Title/Abstract] OR hurricane*[Title/Abstract] OR cyclone*[Title/Abstract] OR "vector borne disease"[Title/Abstract] OR desertification[Title/Abstract] OR deglaciation[Title/Abstract] OR deforestation[Title/Abstract] OR ecomigration[Title/Abstract] OR extinction[Title/Abstract] OR "river disappearance"[Title/Abstract:~2] OR "climate adaptation"[Title/Abstract:~6] OR "climate resilience" [Title/Abstract:~6] OR "climate mitigation"[Title/Abstract:~6] #3 geriatric[Title/Abstract] OR senior*[Title/Abstract] OR elder*[Title/Abstract] OR aged[Title/Abstract] OR ageing[Title/Abstract] OR ((old[Title/Abstract] OR older[Title/Abstract]) AND (people[Title/Abstract] OR adult*[Title/Abstract])) #1 AND #2 AND #3 Filter: English, years 2000 - 2023  **Scopus**  ( TITLE-ABS-KEY  (geriatric OR senior* OR elder* OR aged OR ageing OR ( ( old OR older ) W/1 ( people OR adult* ) ) ) ) AND ( TITLE-ABS-KEY ( "climate change" OR greenhouse OR "global warming" OR ( temperature W/1 ( rise* OR rising ) ) OR "sea level" OR "heat wave" OR ( extreme W/1 ( temperature OR weather OR heat OR cold OR events ) ) OR flood* OR drought* OR "forest fire" OR wildfire OR bushfire OR "natural disaster" OR storm OR typhoon OR hurricane OR cyclone OR "vector borne disease" OR desertification OR deglaciation OR deforestation OR ecomigration OR ( climate W/6 ( resilience OR adaptation OR mitigation ) ) ) ) AND ( TITLE-ABS-KEY ( ( mental W/1 ( health OR illness* OR disorder* ) ) OR "psychiatric disorders" OR "mood disorder*" OR anxi* OR panic OR depress* OR distress OR "post traumatic" OR ptsd OR traumatic OR "bipolar disorder" OR schizophren* OR psychosis OR suicid* OR emotion* OR "eco anxiety" OR ecoanxiety OR grief OR solastalgia OR worry* OR worries OR fear ) ) Filter: English, years 2000 - 2023  **Web of Science (including ProQuest)**  #2 "TS= (geriatric OR senior* OR elder* OR aged OR ageing OR ((old OR older) NEAR/0 (people OR adult*))) #2 "TS=((""climate change"" OR greenhouse OR ""global warming"" OR (temperature* NEAR/0 (rise* OR rising)) OR ""sea level"" OR ""heat wave"" OR (extreme NEAR/0 (temperature* OR weather* OR heat OR cold OR events)) OR flood* OR drought* OR ""forest fire*"" OR wildfire OR bushfire OR ""natural disaster*"" OR storm* OR typhoon* OR hurricane* OR cyclone* OR ""vector borne disease"" OR desertification OR deglaciation OR deforestation OR ecomigration OR extinction OR ""river disappearance"" OR (climate NEAR/6 (adaptation OR resilience OR mitigation)) )) " #3 "TS=((( mental NEAR/0 ( health OR illness OR disorder) ) OR ""psychiatric disorder"" OR ""mood disorder"" OR anxi* OR panic OR depress* OR distress OR ""post traumatic"" OR ptsd OR ""bipolar disorder"" OR schizophren* OR psychosis OR suicid* OR emotion* OR ""eco anxiety"" OR ecoanxiety OR grief OR solastalgia OR worry* OR worries OR fear)) " #1 AND #2 AND #3 Filters: English, years 2000 - 2023, Exclude document types: Retracted Publication or News Item or Book Review or Data Paper or Letter or Meeting Abstract  **GreenFile**  #S1 geriatric OR senior* OR elder* OR aged OR ageing OR ((old OR older) AND (people OR adult*)) #S2 “climate change” OR greenhouse OR "global warming" OR (temperature* AND (rise* OR rising)) OR "sea level" OR "heat wave" OR (extreme AND (temperature* OR weather* OR heat OR cold OR events)) OR flood* OR drought* OR "forest fire*" OR wildfire* OR bushfire* OR "natural disaster*" OR storm* OR typhoon* OR hurricane* OR cyclone* OR "vector borne disease*" OR desertification OR deglaciation OR deforestation OR “river disappearance” OR ecomigration OR adaptation OR mitigation OR resilience #S3 (mental AND ( health OR illness OR disorder) ) OR "psychiatric disorder" OR "mood disorder" OR anxi* OR panic OR depress* OR distress OR "post traumatic" OR ptsd OR "bipolar disorder" OR schizophren* OR psychosis OR suicid* OR emotion* OR ecoanxiety OR grief OR solastalgia OR worry* OR worries OR fear #S1 AND #S2 AND #S3 Filters: Publication year 2000 - 2023  **Cochrane Library**  #1 "climate change" OR greenhouse OR "global warming" OR (temperature* NEXT (rise* OR rising)) OR "sea level" OR "heat wave" OR (extreme NEXT (temperature* OR weather* OR heat OR cold OR events)) OR flood* OR drought* OR (forest NEXT fire*) OR wildfire* OR bushfire* OR (natural NEXT disaster*) OR storm* OR typhoon* OR hurricane* OR cyclone* OR (vector NEXT borne NEXT disease*) OR desertification OR deglaciation OR deforestation OR ecomigration OR extinction OR "river disappearance" OR (climate NEAR adaptation) OR (climate NEAR mitigation) OR (climate NEAR resilience) #2 (mental NEXT (health OR illness OR disorder)) OR "psychiatric disorder" OR "mood disorder" OR anxi* OR panic OR depress* OR distress OR "post traumatic" OR PTSD OR "bipolar disorder" OR schizophren* OR suicid* OR emotion* OR ecoanxiety OR grief OR solastalgia OR worry* OR worries OR fear #3 ((geriatric OR senior* OR elder* OR aged OR ageing OR ((old OR older) AND (people OR adult*)))) #1 AND #2 AND #3 Filters: Publication year 2000 - 2023  **Embase**  #1 geriatric:ab,kw,ti OR senior*:ab,kw,ti OR elder*:ab,kw,ti OR aged:ab,kw,ti OR ageing:ab,kw,ti OR ((old:ab,kw,ti OR older:ab,kw,ti) AND (people:ab,kw,ti OR adult*:ab,kw,ti)) #2 ‘climate change’:ab,kw,ti OR greenhouse:ab,kw,ti OR 'global warming':ab,kw,ti OR (temperature:ab,kw,ti AND (rise*:ab,kw,ti OR rising:ab,kw,ti)) OR 'sea level':ab,kw,ti OR 'heat wave':ab,kw,ti OR heatwave*:ab,kw,ti OR 'extreme temperature*':ab,kw,ti OR 'extreme weather*':ab,kw,ti OR 'extreme heat':ab,kw,ti OR 'extreme cold':ab,kw,ti OR 'extreme events':ab,kw,ti OR flood*:ab,kw,ti OR drought*:ab,kw,ti OR 'forest fire*':ab,kw,ti OR wildfire*:ab,kw,ti OR bushfire*:ab,kw,ti OR 'natural disaster*':ab,kw,ti OR storm*:ab,kw,ti OR typhoon*:ab,kw,ti OR hurricane*:ab,kw,ti OR cyclone*:ab,kw,ti OR 'vector borne disease*':ab,kw,ti OR desertification:ab,kw,ti OR deglaciation:ab,kw,ti OR deforestation:ab,kw,ti OR ecomigration:ab,kw,ti OR exctinction:ab,kw,ti OR ‘river disappearance” OR ((climate NEAR/6 adaptation):ab,kw,ti) OR ((climate NEAR/6 mitigation):ab,kw,ti) OR ((climate NEAR/6 resilience):ab,kw,ti) #3 ‘mental health’:ab,kw,ti OR ‘mental illness’:ab,kw,ti OR ‘mental disorder’:ab,kw,ti OR “psychiactric disorder”:ab,kw,ti OR ‘mood disorder’:ab,kw,ti OR anxi*:ab,kw,ti OR panic:ab,kw,ti OR depress*:ab,kw,ti OR distress:ab,kw,ti OR posttraumatic:ab,kw,ti OR 'post-traumatic':ab,kw,ti OR ptsd:ab,kw,ti OR ‘bipolar disorder’:ab,kw,ti OR schizophren*:ab,kw,ti OR psychosis:ab,kw,ti OR suicid*:ab,kw,ti OR emotion*:ab,kw,ti OR ecoanxiety:ab,kw,ti OR grief:ab,kw,ti OR solastalgia:ab,kw,ti OR worry*:ab,kw,ti OR worries:ab,kw,ti OR fear:ab,kw,ti |

| **Supporting Information Table S1:** *Data Extraction Table Headings* | | | | | | | | | | | | |
| --- | --- | --- | --- | --- | --- | --- | --- | --- | --- | --- | --- | --- |
| Paper details: Author/ Title/ Year | Sample size (in mixed age studies, name whole sample and sample we are looking at) | Participant information: Age (record range, mean and median where possible), other defining characteristics such as nationality, gender etc. | Study location (country/ rural etc) | Type of data collection (e.g. online project, via telephone etc) | Exposure time point: include when data was collected in relation to flooding event. | Details on flooding event e.g. severity + duration of event and impact/ severity e.g. loss of life. | Aims of project | Study design | How they describe mental health concepts/ outcomes (include well-being) | Findings on impact on mental health/ well-being | Results stats (effect size/ means/ standard devs) | Future recommendations/ identified gaps |
|  |  |  |  |  |  |  |  |  |  |  |  |  |

| **Supporting Information Table S2:** *Information about Flooding Events* | | |
| --- | --- | --- |
|  | Flood Details  (How the flood came about) | Flood Damage Reported |
| 1 | Not named in paper | “...the 2010–2011 floods were amongst the worst in the regions’ history.” |
| 2 | Typhoon Morakot struck southern Taiwan | “It was one of the worst disasters to hit Taiwan. Nearly 25,000 residents were forced to evacuate, 408 people went missing, 924 people were injured, and 619 people died. It also caused damage costing more than US$3 billion. Because the damaged area was located near the higher mountains in Taiwan, where its aboriginal people live, access difficulties hindered rescue and reconstruction efforts.”  “Of the sample twelve participants were injured, seven experienced the death of close family members, 25 reported damage to housing, 30 relocated to temporary shelters, 37 assessed health status as worse than others their age.” |
| 3 | Not named in paper | “90-95% residents were forced from homes, cost of clean up exceeded several billion dollars.” |
| 4 | Not named in paper | “Apartments sampled still showed signs of flood damage in January of 1998” (6 months post flood event). |
| 5 | Not named in paper but discusses same event as article 6. | “In June 1981, and again in May 1984, southeastern Kentucky experienced serious and widespread flooding… these were among the most severe ever experienced in the area. They damaged thousands of homes, left hundreds homeless, and caused a total of nearly 30 million dollars in damages.” |
| 6 | In May 1984, a storm system brought tornadoes, strong winds, and extensive flooding to southeastern Kentucky. | “In May 1984, a storm system brought tornadoes, strong winds, and extensive flooding to southeastern Kentucky. Despite the sparse population of the area, more than 6,000 homes were damaged, and more than 5,000 persons were forced out of their homes, with losses totalling over $20 million.” |
| 7 | On Wednesday 15th of November 2017, a very high intensity storm, with cumulative precipitation exceeding 280 mm in 13 h, hit the western part of the region of Attica (Greece) around the SE foothills of Pateras Mt, resulting in a flash flood with catastrophic impact for the towns of Mandra and Nea Peramos. | “Catastrophic impact for both towns, death toll of 24, deadliest flood of the last 40 years in Greece. Hundreds left homeless, severe damage to infrastructure.” |
| 8 | Not named in paper | “More than 200,000 people were affected and 21 were killed. One of the worst floods in decades. In Tumpat there were 26,000 evacuees.” |
| 9 | During the summer of 1993, urban and rural residents in the state of Iowa experienced a series of severe storms. The storms, which lasted for months, brought constant record-breaking rains to nine states throughout the summer. | “One of the worst floods in Iowa's history, entire state declared a disaster area in July 1993. Storms lasted for months and brought constant record-breaking rains to nine states across the summer. Minimal loss of life but extensive and widespread damage and financial loss.” |
| 10 | On the 5th and 6th of September 2008 Morpeth was struck by intensive rainfall. The ground water rose rapidly and the river that flows through the center burst its banks. Consequently, Morpeth was hit by one of its worst floods since 1963. Almost a thousand properties were flooded due to the water rise. | “One of the worst floods since 1963, almost a thousand properties flooded due to the water rise.” |
| 11 | Not named in paper but discusses same event as article 10 | “…the residents of Morpeth were confronted with its worst flood in half a century, which left great material damage”. |
| 12 | Not named in paper but discusses same event as article 10 | “…the residents of Morpeth were confronted with its worst flood in half a century, which left great material damage”. |
